# Supplementary material for: Emergency Department Visits in Children Associated with Exposure to Ambient PM1 within Several Hours
Source: Int J Environ Res Public Health. 2023 Mar 10;20(6):4910. doi: 10.3390/ijerph20064910 (PMC10049417; doi:10.3390/ijerph20064910)
Supplement: Supplementary file 1 [file ijerph-20-04910-s001.zip › ijerph-2238035-supplementary.pdf]

Supplementary Material

Emergency Department Visits in Children Associated with Exposure to Ambient PM1 within Several Hours

Yachen Li †, Lifeng Zhu †, Yaqi Wang, Ziqing Tang, Yuqian Huang, Yixiang Wang, Jingjing Zhang and Yunquan Zhang *

Laboratory of Occupational Hazard Identification and Control, Institute of Social Development and Health Management, Hubei Province Key, School of Public Health, Wuhan University of Science and Technology, Wuhan 430065, China

* Correspondence: yunquanzhang@wust.edu.cn

† These authors contributed equally to this work.

Table of Contents

Figure S1 Spearman correlation matrix between ambient air pollutants and meteorological factors in Guangzhou and Shenzhen, China, 2015–2016.

Table S1 ORs (95% CIs) for PEDVs in Guangzhou at various exposure hours associated with per IQR increase in PM_1, $PM_{2.5}$, and per 10% increase in $PM_1/PM_{2.5}$ ratio.

Table S2 ORs (95% CIs) for PEDVs in Shenzhen at various exposure hours associated with per IQR increase in PM_1, $PM_{2.5}$, and per 10% increase in $PM_1/PM_{2.5}$ ratio.

Table S3 Sensitive analysis of PM-PEDVs associations in Guangzhou and Shenzhen by changing modelling choices of conditional logistic regression.

Table S4 Season-specific ORs (95% CIs) for PEDVs in Guangzhou at different lag periods associated with per IQR increase in PM_1, $PM_{2.5}$, and per 10% increase in $PM_1/PM_{2.5}$ ratio.

Table S5 Season-specific ORs (95% Cis) for PEDVs in Shenzhen at different lag periods associated with per IQR increase in PM_1, $PM_{2.5}$, and per 10% increase in $PM_1/PM_{2.5}$ ratio.

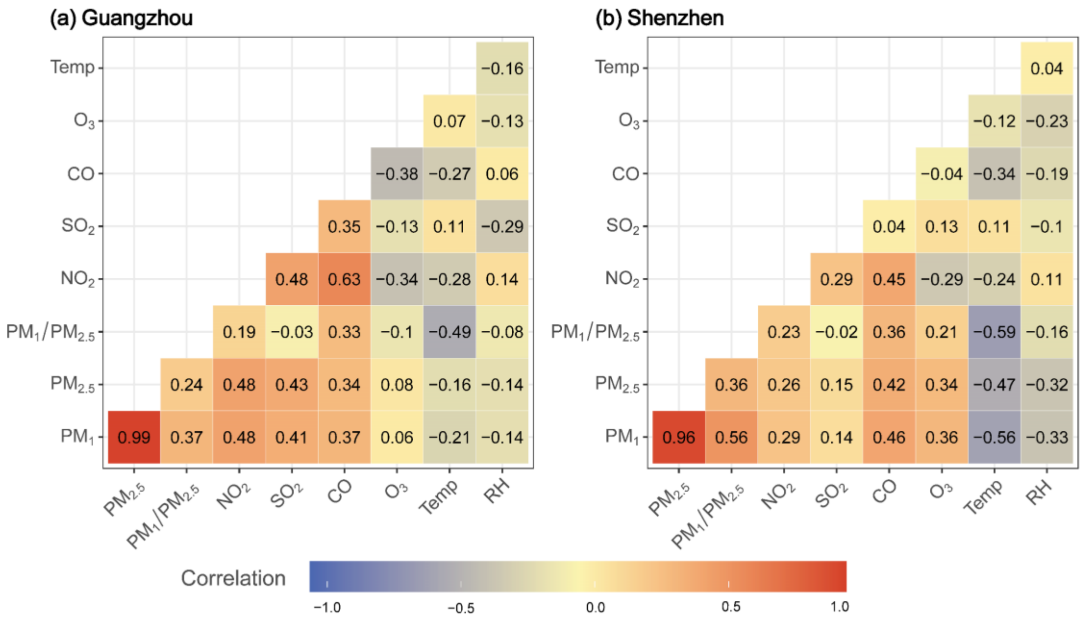

Figure S1. Spearman correlation matrix between ambient air pollutants and meteorological factors in Guangzhou and Shenzhen, China, 2015–2016. Abbreviations: PM_1, particulate matter with aerodynamic diameter ≤1 µm; $PM_{2.5}$, particulate matter with aerodynamic diameter ≤2.5 µm; NO_2, nitrogen dioxide; SO_2, sulfur dioxide; O_3, ozone; CO, carbon monoxide; Temp, temperature; RH, relative humidity.

Table S1. ORs (95% CIs) for PEDVs in Guangzhou at various exposure hours associated with per IQR increase in PM_1, $PM_{2.5}$, and per 10% increase in $PM_1/PM_{2.5}$ ratio.

Pollutant	Lag	OR [95% CI]	*P*-value
PM_1			
	0–3 h	1.039 (1.027–1.050)	<0.001
	4–6 h	1.045 (1.033–1.057)	<0.001
	7–12 h	1.043 (1.031–1.055)	<0.001
	13–24 h	1.029 (1.016–1.042)	<0.001
	25–48 h	1.010 (0.997–1.024)	0.137
	49–72 h	1.001 (0.988–1.015)	0.844
	73–96 h	0.991 (0.978–1.004)	0.163
$PM_{2.5}$			
	0–3 h	1.040 (1.028–1.051)	<0.001
	4–6 h	1.046 (1.035–1.058)	<0.001
	7–12 h	1.043 (1.031–1.055)	<0.001
	13–24 h	1.029 (1.016–1.041)	<0.001
	25–48 h	1.010 (0.997–1.023)	0.136
	49–72 h	1.000 (0.987–1.013)	0.951
	73–96 h	0.988 (0.976–1.001)	0.070
$PM_1/PM_{2.5}$			
	0–3 h	0.992 (0.981–1.004)	0.191
	4–6 h	0.992 (0.981–1.003)	0.162
	7–12 h	0.998 (0.987–1.009)	0.752
	13–24 h	1.008 (0.995–1.021)	0.256
	25–48 h	1.004 (0.990–1.018)	0.566
	49–72 h	1.026 (1.011–1.040)	<0.001
	73–96 h	1.026 (1.012–1.040)	<0.001

Abbreviations: CI, confidence interval; OR, odds ratio; PEDVs, pediatric emergency department visits; PM_1, particulate matter with aerodynamic diameter ≤1 μm; $PM_{2.5}$, particulate matter with aerodynamic diameter ≤2.5 μm.

Table S2. ORs (95% Cis) for PEDVs in Shenzhen at various exposure hours associated with per IQR increase in PM_1, $PM_{2.5}$, and per 10% increase in $PM_1/PM_{2.5}$ ratio.

Pollutant	Lag	OR [95% CI]	*P*-value
PM_1			
	0–3 h	1.032 (1.019–1.044)	<0.001
	4–6 h	1.041 (1.028–1.053)	<0.001
	7–12 h	1.043 (1.030–1.057)	<0.001
	13–24 h	1.023 (1.009–1.037)	0.001
	25–48 h	1.031 (1.016–1.047)	<0.001
	49–72 h	1.019 (1.004–1.035)	0.013
	73–96 h	1.004 (0.990–1.020)	0.559
$PM_{2.5}$			
	0–3 h	1.027 (1.016–1.039)	<0.001
	4–6 h	1.035 (1.024–1.046)	<0.001
	7–12 h	1.037 (1.025–1.049)	<0.001
	13–24 h	1.018 (1.006–1.031)	0.005
	25–48 h	1.024 (1.010–1.038)	<0.001
	49–72 h	1.014 (1.000–1.028)	0.050
	73–96 h	1.004 (0.990–1.017)	0.613
$PM_1/PM_{2.5}$			
	0–3 h	1.012 (1.004–1.020)	0.005
	4–6 h	1.014 (1.005–1.022)	0.001
	7–12 h	1.016 (1.008–1.025)	<0.001
	13–24 h	1.017 (1.008–1.026)	<0.001
	25–48 h	1.019 (1.010–1.029)	<0.001
	49–72 h	1.012 (1.003–1.022)	0.012
	73–96 h	1.003 (0.994–1.012)	0.534

Abbreviations: CI, confidence interval; OR, odds ratio; PEDVs, pediatric emergency department visits; PM_1, particulate matter with aerodynamic diameter ≤1 µm; $PM_{2.5}$, particulate matter with aerodynamic diameter ≤2.5 µm.

Table S3. Sensitive analysis of PM-PEDVs associations in Guangzhou and Shenzhen by changing modelling choices of conditional logistic regression.

Models	OR (95% CI) per IQR increase in PM_1 and $PM_{2.5}$			
	Guangzhou		Shenzhen	
	OR (95% CI) for PM_1	OR (95% CI) for $PM_{2.5}$	OR (95% CI) for PM_1	OR (95% CI) for $PM_{2.5}$
**Single-pollutant**	1.045 (1.033–1.057)	1.046 (1.035–1.058)	1.041 (1.028–1.053)	1.035 (1.024–1.046)
**Bi-pollutant**				
+ NO_2	1.038 (1.024–1.052)	1.040 (1.026–1.054)	1.034 (1.020–1.048)	1.028 (1.016–1.041)
+ SO_2	1.027 (1.012–1.042)	1.030 (1.015–1.045)	1.037 (1.023–1.051)	1.031 (1.018–1.044)
+ CO	1.041 (1.028–1.055)	1.043 (1.030–1.056)	1.048 (1.034–1.062)	1.040 (1.027–1.052)
+ O_3	1.045 (1.032–1.057)	1.046 (1.034–1.058)	1.040 (1.027–1.054)	1.034 (1.021–1.046)
**Tri-pollutant**				
+NO_2+SO_2	1.025 (1.009–1.041)	1.028 (1.012–1.044)	1.033 (1.019–1.048)	1.027 (1.014–1.040)
+NO_2+CO	1.027 (1.012–1.043)	1.030 (1.015–1.045)	1.043 (1.028–1.058)	1.035 (1.022–1.048)
+NO_2+O_3	1.020 (1.004–1.036)	1.023 (1.007–1.039)	1.034 (1.018–1.049)	1.027 (1.013–1.041)
+SO_2+CO	1.034 (1.019–1.049)	1.037 (1.022–1.051)	1.041 (1.026–1.056)	1.033 (1.020–1.046)
+SO_2+O_3	1.036 (1.021–1.050)	1.038 (1.024–1.052)	1.034 (1.020–1.048)	1.028 (1.015–1.041)
+CO+O_3	1.038 (1.023–1.052)	1.039 (1.026–1.053)	1.047 (1.033–1.062)	1.039 (1.025–1.052)
**Degree of freedom**				
df (Temp)=4	1.045 (1.033–1.057)	1.046 (1.035–1.058)	1.041 (1.028–1.053)	1.035 (1.024–1.047)
df (Temp)=5	1.045 (1.033–1.057)	1.046 (1.035–1.058)	1.041 (1.028–1.053)	1.035 (1.024–1.046)
df (Temp)=6	1.045 (1.033–1.056)	1.046 (1.035–1.058)	1.041 (1.029–1.054)	1.035 (1.024–1.047)
df (RH)=4	1.044 (1.033–1.056)	1.046 (1.034–1.057)	1.041 (1.028–1.054)	1.035 (1.024–1.047)
df (RH)=5	1.044 (1.033–1.056)	1.046 (1.035–1.057)	1.041 (1.028–1.054)	1.035 (1.024–1.047)
df (RH)=6	1.044 (1.032–1.056)	1.046 (1.034–1.057)	1.041 (1.029–1.054)	1.035 (1.024–1.047)
**Exposure time**				
Temp (0–2 h)	1.045 (1.033–1.056)	1.046 (1.034–1.057)	1.041 (1.029–1.054)	1.035 (1.024–1.047)

Temp (0–7 h)	1.044 (1.032–1.055)	1.044 (1.033–1.056)	1.043 (1.030–1.055)	1.036 (1.025–1.048)
RH (0–2 h)	1.044 (1.033–1.056)	1.045 (1.034–1.057)	1.039 (1.026–1.052)	1.033 (1.022–1.045)
RH (0–7 h)	1.042 (1.031–1.054)	1.043 (1.032–1.055)	1.036 (1.024–1.049)	1.031 (1.020–1.043)

Abbreviations: CI, confidence interval; OR, odds ratio; PEDVs, pediatric emergency department visits; PM_1, particulate matter with aerodynamic diameter ≤1 μm; $PM_{2.5}$, particulate matter with aerodynamic diameter ≤2.5 μm; SO_2, sulfur dioxide; NO_2, nitrogen dioxide; CO, carbon monoxide; O_3, ozone; Temp, temperature; RH, relative humidity; df, degree of freedom.

Table S4. Season–specific ORs (95% CIs) for PEDVs in Guangzhou at different lag periods associated with per IQR increase in PM_1, $PM_{2.5}$, and per 10% increase in $PM_1/PM_{2.5}$ ratio.

Lag	Exposure	Season	OR [95% CI]	P for heterogeneity
0–3 h				
	PM_1	Cold	1.068 (1.053–1.084)	0.004
		Warm	1.036 (1.021–1.052)	
	$PM_{2.5}$	Cold	1.072 (1.057–1.088)	0.004
		Warm	1.039 (1.024–1.055)	
	$PM_1/PM_{2.5}$	Cold	0.964 (0.938–0.990)	0.138
		Warm	0.986 (0.973–1.000)	
4–6 h				
	PM_1	Cold	1.072 (1.056–1.088)	0.014
		Warm	1.045 (1.030–1.060)	
	$PM_{2.5}$	Cold	1.075 (1.059–1.091)	0.026
		Warm	1.050 (1.035–1.065)	
	$PM_1/PM_{2.5}$	Cold	0.968 (0.943–0.994)	0.273
		Warm	0.984 (0.972–0.997)	
7–12 h				
	PM_1	Cold	1.077 (1.059–1.094)	0.003
		Warm	1.042 (1.028–1.058)	
	$PM_{2.5}$	Cold	1.077 (1.060–1.095)	0.011
		Warm	1.048 (1.032–1.063)	
	$PM_1/PM_{2.5}$	Cold	0.973 (0.947–0.999)	0.198
		Warm	0.992 (0.979–1.005)	
13–24 h				
	PM_1	Cold	1.068 (1.050–1.087)	0.001
		Warm	1.028 (1.012–1.044)	
	$PM_{2.5}$	Cold	1.069 (1.051–1.087)	0.003
		Warm	1.032 (1.016–1.048)	
	$PM_1/PM_{2.5}$	Cold	1.013 (0.983–1.044)	0.192
		Warm	0.991 (0.976–1.006)	
25–48 h				
	PM_1	Cold	1.049 (1.030–1.069)	0.006
		Warm	1.014 (0.998–1.030)	
	$PM_{2.5}$	Cold	1.048 (1.028–1.068)	0.027
		Warm	1.019 (1.003–1.035)	
	$PM_1/PM_{2.5}$	Cold	1.032 (0.998–1.067)	0.002
		Warm	0.974 (0.959–0.990)	
49–72 h				
	PM_1	Cold	1.029 (1.009–1.049)	0.124
		Warm	1.009 (0.994–1.024)	
	$PM_{2.5}$	Cold	1.027 (1.007–1.047)	0.215
		Warm	1.011 (0.996–1.026)	

Table S4. (continued)

Lag	Exposure	Season	OR [95% CI]	P for heterogeneity
73–96 h	$PM_1/PM_{2.5}$	Cold	1.044 (1.009–1.080)	0.025
		Warm	1.000 (0.984–1.017)	
	PM_1	Cold	1.006 (0.987–1.026)	0.308
		Warm	0.994 (0.978–1.009)	
	$PM_{2.5}$	Cold	1.003 (0.984–1.023)	0.438
		Warm	0.994 (0.978–1.009)	
	$PM_1/PM_{2.5}$	Cold	1.034 (1.000–1.068)	0.174
		Warm	1.008 (0.991–1.024)	

Abbreviations: CI, confidence interval; OR, odds ratio; PEDVs, pediatric emergency department visits; PM_1, particulate matter with aerodynamic diameter ≤1 µm; $PM_{2.5}$, particulate matter with aerodynamic diameter ≤2.5 µm; Warm season, April to September; Cold season, October to March of the next year.

Table S5. Season–specific ORs (95% Cis) for PEDVs in Shenzhen at different lag periods associated with per IQR increase in PM_1, $PM_{2.5}$, and per 10% increase in $PM_1/PM_{2.5}$ ratio.

Lag	Exposure	Season	OR (95% CI)	P for heterogeneity
0–3 h				
	PM_1	Cold	1.037 (1.021–1.054)	0.011
		Warm	1.010 (0.998–1.023)	
	$PM_{2.5}$	Cold	1.034 (1.019–1.051)	0.025
		Warm	1.011 (0.998–1.024)	
	$PM_1/PM_{2.5}$	Cold	1.016 (0.996–1.037)	0.379
		Warm	1.006 (0.997–1.015)	
4–6 h				
	PM_1	Cold	1.041 (1.024–1.057)	0.082
		Warm	1.022 (1.009–1.035)	
	$PM_{2.5}$	Cold	1.038 (1.022–1.054)	0.124
		Warm	1.022 (1.009–1.035)	
	$PM_1/PM_{2.5}$	Cold	1.013 (0.993–1.033)	0.771
		Warm	1.009 (1.000–1.019)	
7–12 h				
	PM_1	Cold	1.043 (1.026–1.060)	0.079
		Warm	1.023 (1.009–1.037)	
	$PM_{2.5}$	Cold	1.040 (1.023–1.057)	0.087
		Warm	1.021 (1.007–1.035)	
	$PM_1/PM_{2.5}$	Cold	1.016 (0.995–1.037)	0.810
		Warm	1.013 (1.003–1.023)	
13–24 h				
	PM_1	Cold	1.031 (1.012–1.050)	0.017
		Warm	1.003 (0.989–1.016)	
	$PM_{2.5}$	Cold	1.029 (1.011–1.048)	0.014
		Warm	1.001 (0.987–1.014)	
	$PM_1/PM_{2.5}$	Cold	1.016 (0.995–1.038)	0.817
		Warm	1.013 (1.003–1.023)	
25–48 h				
	PM_1	Cold	1.038 (1.016–1.061)	0.033
		Warm	1.010 (0.996–1.024)	
	$PM_{2.5}$	Cold	1.037 (1.016–1.059)	0.015
		Warm	1.005 (0.991–1.020)	
	$PM_1/PM_{2.5}$	Cold	1.011 (0.987–1.035)	0.599
		Warm	1.018 (1.007–1.028)	
49–72 h				
	PM_1	Cold	1.024 (1.001–1.048)	0.135
		Warm	1.004 (0.990–1.018)	
	$PM_{2.5}$	Cold	1.023 (1.001–1.046)	0.094
		Warm	1.001 (0.987–1.014)	

Table S5. (continued)

Lag	Exposure	Season	OR (95% CI)	P for heterogeneity
	$PM_1/PM_{2.5}$	Cold	1.015 (0.991–1.040)	0.576
		Warm	1.008 (0.997–1.018)	
73–96 h				
	PM_1	Cold	1.024 (1.001–1.048)	0.135
		Warm	1.004 (0.990–1.018)	
	$PM_{2.5}$	Cold	1.023 (1.001–1.046)	0.094
		Warm	1.001 (0.987–1.014)	
	$PM_1/PM_{2.5}$	Cold	1.015 (0.991–1.040)	0.576
		Warm	1.008 (0.997–1.018)	

Abbreviations: CI, confidence interval; OR, odds ratio; PEDVs, pediatric emergency department visits; PM_1, particulate matter with aerodynamic diameter ≤1 μm; $PM_{2.5}$, particulate matter with aerodynamic diameter ≤2.5 μm; Warm season, April to September; Cold season, October to March of the next year.
